# Wire ‘missing’: a rare presentation of preoperative localization wire system dislocation

**DOI:** 10.1186/s13019-014-0162-0

**Published:** 2014-09-30

**Authors:** Xiaofeng Chen, Shaohua Wang, Zhenhua Hao, Qinyun Ma

**Affiliations:** Department of Thoracic Surgery, Huashan Hospital, Fudan University, No 12, Middle Urumqi Rd, Shanghai, 200040 China

**Keywords:** Lung resection, Video-assisted thoracoscopic surgery, Computed tomography

## Abstract

A full account is presented of a 53-year-old Chinese woman with a small subpleura nodule in the right upper lobe, which was successfully localized by a Dualok-wire system under computed tomography guidance preoperatively. However, during operation, the hook wire was ‘missing’, neither in the thorax, nor in the lung. With the help of bed-side chest film, the missing wire, which was twisted in the patient's chest wall, was localized and removed through another small incision. Posterior segmentectomy was administered, and the pulmonary lesion was pathologically diagnosed as atypical alveolar hyperplasia.

**Electronic supplementary material:**

The online version of this article (doi:10.1186/s13019-014-0162-0) contains supplementary material, which is available to authorized users.

## Background

With the development of imaging technique and mini-invasive surgical technique, there are more and more chances for thoracic surgeons to tackle with small pulmonary nodules and small ground glass opacities (GGO). Preoperative computed tomography (CT)-guided localization of small pulmonary lesions by various types of wire systems is demonstrated to be effective and safe [1],[2], with minor complications, such as mild pneumothorax, intrapulmonary bleeding and wire dislocation. Here, we report a rare case of wire dislocation presenting as wire ‘missing’ during video-assisted thoracoscopic surgery (VATS). The wire, which was found by the bed-side X-ray twisted in the chest wall, was finally taken out through another skin incision.

## Case presentation

**Figure 1:**
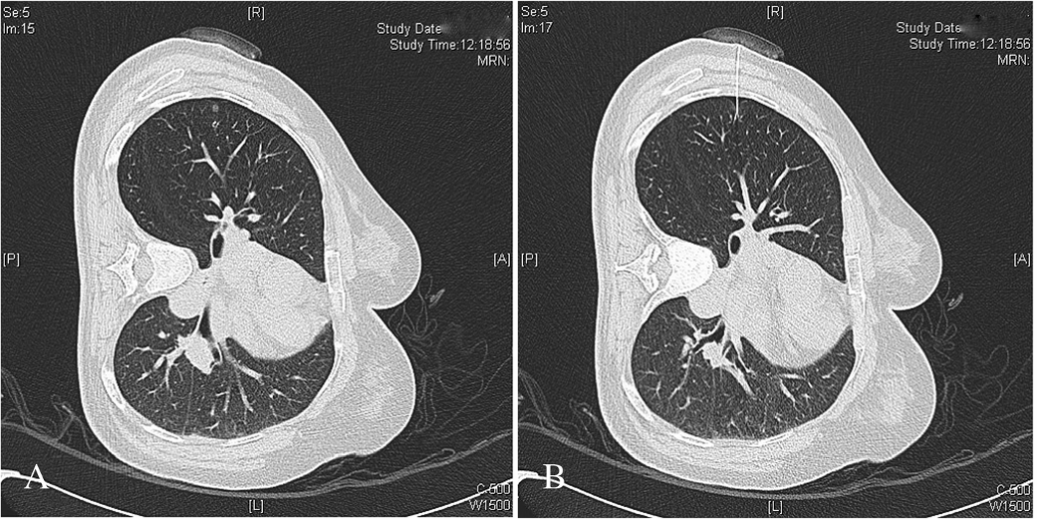
**CT images during localization. (A)** CT image showing the subpleura pulmonary lesion in the S2b of the right upper lobe; **(B)** CT image showing mild pneumothorax and the tip of wire 4 mm below the lesion.

A 53-year-old Chinese woman was referred to our hospital for a 6.0-mm subpleural nodule in S2b of the right upper pulmonary lobe in chest CT images (Figure 1A). Preoperative blood examination revealed no abnormality, and cardiopulmonary examination showed the patient was able to tolerate the single-lung ventilation and pulmonary resection. To aid in the diagnosis, a video-assisted thoracoscopic pulmonary wedge resection was scheduled, and a preoperative CT-guided transthoracic localization procedure was used to guide the staple line during operation and facilitate finding the lesion in the specimen.

On the day of the operation, CT-guided marking for the right pulmonary lesion was performed before the patient was sent to the operating room. The procedure was performed in the left-sided lateral position with a Bard Dualok breast lesion localization wire (gauge size and needle length: 20 g*10.7 cm, Bard Peripheral Vascular, Inc, AZ, USA). The patient was stable during the procedure, and post-procedure CT showed the wire tip 4 mm below the lesion and mild pneumothorax (Figure 1B). The result was acceptable and the patient was transferred to the operation room.

**Figure 2:**
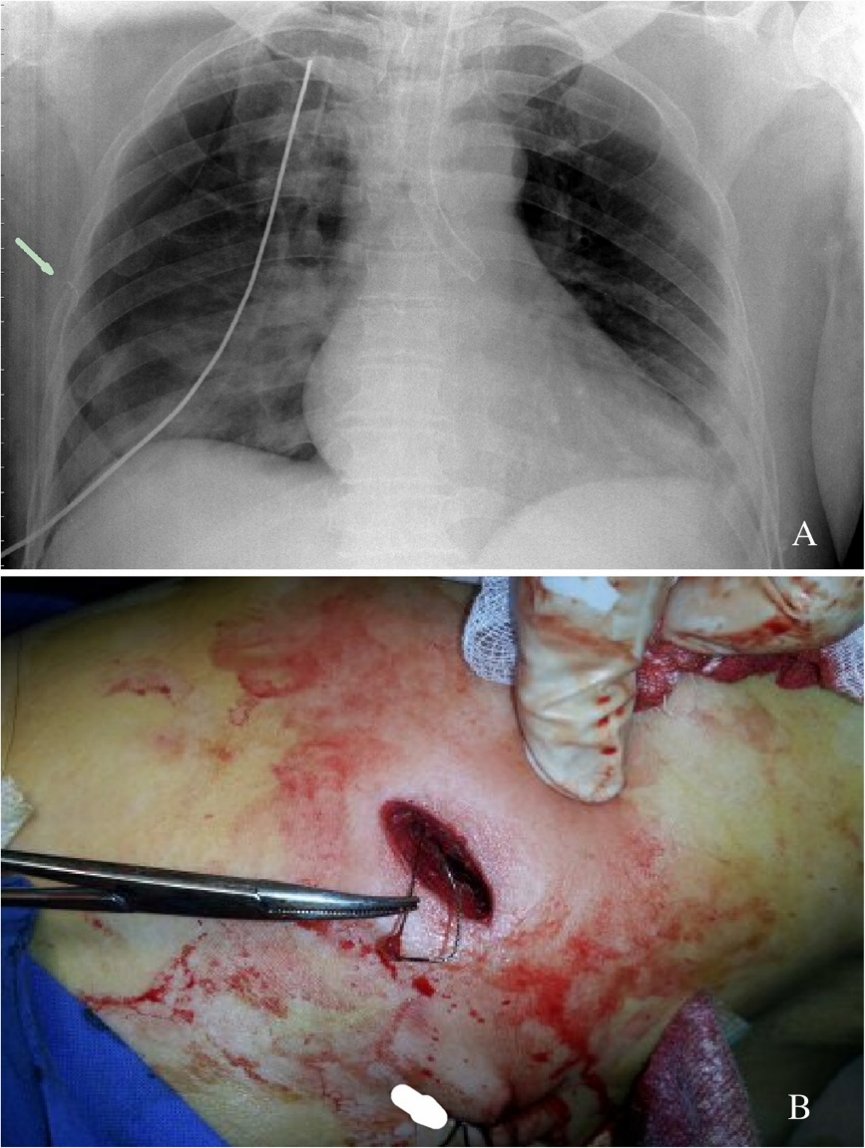
**The 'missing' wire was localized and removed. (A)** Bed-side X-ray showing the wire (arrow) twisted in the lateral chest wall; **(B)** The wire was removed through another incision just at the point of puncture posterior to the manipulation hole (arrow).

The operation began 35 min later, and on the thoracoscopic exploration, we were surprised that the localization wire was missing, neither in the thoracic cavity, nor in the pulmonary parenchymal on palpation through the manipulation hole. There was a red eyelet on both the visceral pleura in the right S2 and the correspondent parietal pleura, where the wire was punctured. The surgeon inserted his finger through the manipulation hole to palpate the red eyelet on the interior surface of the chest wall but failed to feel the wire. Posterior segmentectomy was performed. The lesion was found to be atypical alveolar hyperplasia (AAH) by frozen examination but the wire was not in the specimen yet. We asked the bed-side X-ray for help, which showed that the wire was twisted in the chest wall (Figure 2A). Then we made another incision just at the point of puncture and took the wire out (Figure 2B), during which the intercostal vessel was injured by the twisted hook and ligated for hemostasis. The postoperative course was uneventful.

### Discussion

Recently, opportunities of locating small lesions in the lung and of performing video-assisted thoracoscopic surgery procedures have increased. Accordingly, some preoperative marking under CT guidance for small lesions are considered using methylene blue [3] or various types of wire devices [1],[2]. Although pneumothorax and dislocation are most encountered complications, such kind of dislocation as described in our case is extremely rare. The possible explanation, we think, is that the lung tissue hooked by the wire was not enough due to the fact that the lesion was too superficial. Therefore, the wire dehooked the lung and was twisted by the muscles during respiration and movement.

To avert such dislocation, we make suggestions as follows: (1) the operation should be performed just after the localization procedure to reduce the risk of dislocation caused by patient's movement; (2) it is strongly advisable that the tip of the wire slighterly deeper to the lesion than usual, especially for subpleural nodules, for the distance between the hook wire tip and pleural surface was demonstrated to be the major significant factor for successful computed tomography-guided nodule localization for subsequent video-assisted thoracic surgery resection by multivariate analysis [4]; (3) Other methods without hooking pulmonary tissue [5] could be tried for such superficial subpleural nodules.

In case of occurrence of such kind of dislocation, the wire can be removed through a skin incision or by VATS. For our case, the point of puncture was near the manipulation hole, we think it was easier to remove it through another small incision. Due to the hook-shaped tip of the wire, it is more likely that the tip hooked intercostal vessels, increasing the risk of bleeding. Great attention should be paid to avoiding injury to the intercostal vessels during removal of dislocated wire.

Last but not least, with the popularity of low-dose thin-section CT screening, peripheral pulmonary sub-solid nodules were detected with increasing number. As for the surgical indication, current available evidence [6] recommends at least 3-month follow-up for pure GGO ≥ 5 mm in size, followed by yearly CT scan for at least 3 years. A 6 mm pure GGO, as in this case, is most likely to be a preinvasive AAH or adenocarcinoma in situ (AIS), which is amenable to a conservative approach with CT surveillance. The futile operation in this case resulted in the ‘wire missing episode’, at the expense of the risks the patient had to go through. Therefore, one should always bears in mind that minimally invasive surgery does not mean minimal risk, and thus, should be reserved only for those who are indeed expected to benefit from the procedure.

## Conclusion

We have reported a case of wire dislocation with rare presentation and its treatment, not only hoping to add to the experience of preoperative localization of small lung lesions and video-assisted thoracoscopic lung resection, but also warning against the over-treatment tendency in face of increasing detected small pulmonary lesions by CT screenings.

## Consent

Written informed consent was obtained from the patient for publication of this case report and any accompanying images. A copy of the written consent is available for review by the Editor-in-Chief of this journal.

## Authors' contributions

Chen X performed the surgery, Hao Z assisted in the surgery, Ma Q collected the images and Wang S wrote the report. All authors read and approved the final manuscript.

## Authors’ original submitted files for images

Below are the links to the authors’ original submitted files for images. Authors’ original file for figure 1 Authors’ original file for figure 2

## Abbreviation

VATS: Video-assisted thoracoscopic surgery
CT: Computed tomography
AAH: Atypical adenomatous hyperplasia
AIS: Adenocarcinoma in situ
GGO: Ground glass opacities
