# Supplementary figures and images for: Wire ‘missing’: a rare presentation of preoperative localization wire system dislocation
Source: J Cardiothorac Surg. 2014 Sep 30;9:162. doi: 10.1186/s13019-014-0162-0 (PMC4189601; doi:10.1186/s13019-014-0162-0)

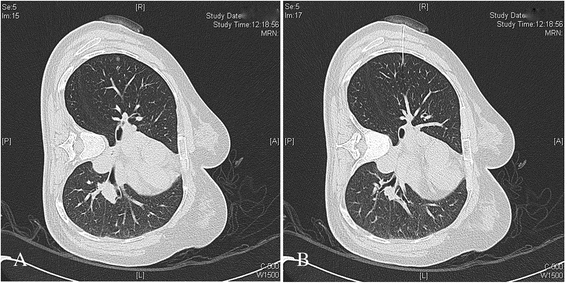

Supplement: Supplementary file 1 — Authors’ original file for figure 1 [file 13019_2014_162_MOESM1_ESM.gif]

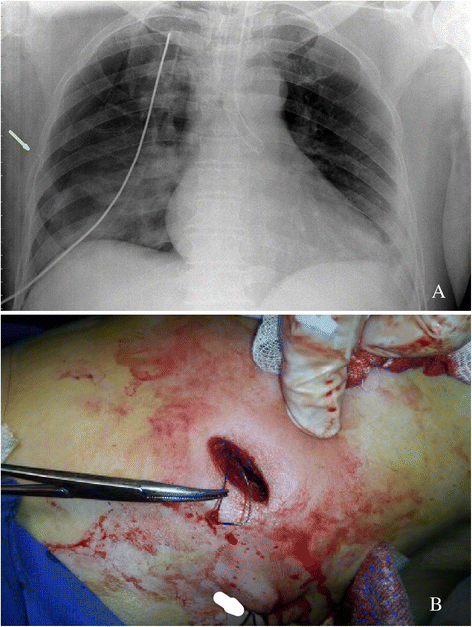

Supplement: Supplementary file 2 — Authors’ original file for figure 2 [file 13019_2014_162_MOESM2_ESM.gif]
